# *Chlamydomonas* Flavodiiron Proteins Facilitate Acclimation to Anoxia During Sulfur Deprivation

**DOI:** 10.1093/pcp/pcv085

**Published:** 2015-06-10

**Authors:** Martina Jokel, Sergey Kosourov, Natalia Battchikova, Anatoly A. Tsygankov, Eva Mari Aro, Yagut Allahverdiyeva

**Affiliations:** ^1^Laboratory of Molecular Plant Biology, Department of Biochemistry, University of Turku, FI-20014 Turku, Finland; ^2^Institute of Basic Biological Problems, RAS, Pushchino, 142290 Russia

**Keywords:** Alternative electron transport, *Chlamydomonas reinhardtii*, Flavodiiron proteins, O_2_ photoreduction, Photosynthesis, Sulfur deprivation

## Abstract

The flavodiiron proteins (FDPs) are involved in the detoxification of oxidative compounds, such as nitric oxide (NO) or O_2_ in Archaea and Bacteria. In cyanobacteria, the FDPs Flv1 and Flv3 are essential in the light-dependent reduction of O_2_ downstream of PSI. Phylogenetic analysis revealed that two genes (*flvA* and *flvB*) in the genome of *Chlamydomonas reinhardtii* show high homology to *flv1* and *flv3* genes of the cyanobacterium *Synechocystis* sp. PCC 6803. The physiological role of these FDPs in eukaryotic green algae is not known, but it is of a special interest since these phototrophic organisms perform oxygenic photosynthesis similar to higher plants, which do not possess FDP homologs. We have analyzed the levels of *flvA* and *flvB* transcripts in *C. reinhardtii* cells under various environmental conditions and showed that these genes are highly expressed under ambient CO_2_ levels and during the early phase of acclimation to sulfur deprivation, just before the onset of anaerobiosis and the induction of efficient H_2_ photoproduction. Importantly, the increase in transcript levels of the *flvA* and *flvB* genes was also corroborated by protein levels. These results strongly suggest the involvement of FLVA and FLVB proteins in alternative electron transport.

## Introduction

*Chlamydomonas reinhardtii* is a soil-dwelling green alga with great flexibility in its photosynthetic machinery and metabolism, which are employed to cope with changing light, carbon and nutrient supplies and oxic/anoxic conditions. During photosynthesis, specialized antenna complexes harvest and transfer light energy to the PSII and PSI reaction centers, where primary charge separation initiates photosynthetic linear electron flow by oxidizing water at PSII and reducing NADP^+^ to NADPH downstream of PSI. These electron transfer reactions are coupled with proton pumping across the thylakoid membrane, and the resulting proton gradient, ΔpH, drives the ATP synthesis. Photosynthetic organisms have developed different photoprotective mechanisms and alternative electron transport pathways to prevent the over-reduction of the photosynthetic electron transport chain and to maintain an optimal NAD(P)H/ATP ratio under different environmental conditions (reviewed in Peltier et al. 2010, Cardol et al. 2011, Shikanai 2014).

In cyanobacteria, flavodiiron proteins (FDPs, also called A-type flavoproteins, Flvs) function as a strong electron sink, redirecting excess electrons to O_2_ in a non-harmful way (reviewed in Allahverdiyeva et al. 2015a, Allahverdiyeva et al. 2015b). Since *C. reinhardtii* possesses two genes with high homology to *Synechocystis* sp. strain PCC 6803 (hereafter, *Synechocystis*) *flv* genes, it is highly conceivable that the proteins encoded by these genes are also involved in photosynthetic electron transport in *C. reinhardtii*.

FDPs are a family of enzymes with nitric oxide (NO)/O_2_-reductase activity and have a modular structure with a N-terminal metallo-β-lactamase-like domain and a C-terminal flavodoxin-like domain as core units (Vicente et al. 2002). The metallo-β-lactamase module harbors a non-heme di-iron center with histidine and carboxylate residues as ligands; this is the active site of NO/O_2_ reduction (Silaghi-Dumitrescu et al. 2003). At the C-terminus, the FMN prosthetic group is embedded and acts as the electron donor for the di-iron domain. In FDP monomers, these two redox centers are too distant from each other to perform electron transfer (Vicente et al. 2008). However, the monomers can build a ‘head-to-tail’ dimer structure for efficient electron transfer. This arrangement brings the di-iron center of each monomer in close contact with the FMN moiety from the other monomer (Vicente et al. 2008).

In organisms that conduct oxygenic photosynthesis, including cyanobacteria, green algae, mosses and lycophytes, an additional NAD(P)H:flavinoxidoreductase module is fused at the C-terminus of the FDPs. These oxygenic photosynthetic organisms always possess at least two different FDPs, which are grouped into the two clusters A and B (Zhang et al. 2009). It is noteworthy that genes encoding FDP homologs have not been detected in the sequenced genomes of diatoms, haptophytes or higher plants, *Picea sitchensis* being an exception. An ancient plant, *P. sitchensis* possesses a single gene with homology to *flv*; however, the enzyme encoded by this gene lacks the additional C-terminal domain that is typical of all other oxygenic photosynthetic organisms (Allahverdiyeva et al. 2015a).

Most studies conducted so far on the function of FDPs in photosynthetic organisms have been focused on cyanobacteria. The genome of *Synechocystis,* a non-N_2_-fixing, unicellular cyanobacterium, contains four genes (*sll1521*, *sll0219*, *sll0550* and *sll0217*) encoding a family of FDPs: Flv1, Flv2, Flv3 and Flv4, respectively. A reverse genetics approach applied to *Synechocystis* has demonstrated the essential function of Flv1 and Flv3 proteins in the light-dependent reduction of O_2_, also known as the Mehler-like reaction (Helman et al. 2003). Recently, it has been found that Flv1 and Flv3 proteins are crucial for safeguarding the photosynthetic apparatus, particularly the PSI complex, under fluctuating light intensities, mimicking natural light conditions (Allahverdiyeva et al. 2013, Allahverdiyeva et al. 2015b). The other two FDPs, Flv2 and Flv4, are not involved in O_2_ photoreduction (Helman et al. 2003, Allahverdiyeva et al. 2015a). Instead, these proteins function as a heterodimer in the photoprotection of PSII under CO_2_-limiting and high light conditions by releasing excess excitation pressure at the acceptor side of PSII to a currently unknown electron acceptor (Zhang et al. 2009, Zhang et al. 2012), in co-operation with phycobilisomes (Bersanini et al. 2014, Chukhutsina et al. 2015).

The filamentous heterocystous N_2_-fixing cyanobacterium, *Anabaena* sp. strain PCC 7120 (hereafter *Anabaena*), possesses six FDPs. Flv1A and Flv3A proteins are specific to vegetative cells and probably function in the Mehler-like reaction, whereas Flv2 and Flv4 proteins presumably mediate photoprotection of PSII, similar to their role in *Synechocystis* (Ermakova et al. 2013, Ermakova et al. 2014). The additional set of two FDPs in *Anabaena*, Flv1B and Flv3B, are heterocyst specific (Ermakova et al. 2013). It has been shown that Flv3B protects nitrogenase by performing light-induced O_2_ uptake and maintaining micro-oxic conditions inside of the heterocysts, while the role of Flv1B remains unknown (Ermakova et al. 2014).

In the eukaryotic green alga *C. reinhardtii*, two *flv* genes have been identified as paralogs in each cluster: *flvA* (Cre12.g531900) and *flvB* (Cre16.g691800). Despite a lack of sufficient experimental data, the high homology between the cyanobacterial and algal FDP proteins makes the involvement of FDPs in O_2_ photoreduction highly likely (Zhang et al. 2009, Peltier et al. 2010, Cardol et al. 2011, Dang et al. 2014).

In this work, we analyzed the expression patterns of *C. reinhardtii flvA* and *flvB* at the transcript and protein levels under different environmental conditions, including acclimation to different light intensities, CO_2_ concentrations and sulfur deprivation. Our results strongly support the involvement of the FLVA and FLVB proteins in alternative electron transfer.

## Results

### Selection of the appropriate reference genes

Before analyzing the transcript level of *flvA* and *flvB* with real-time quantitative reverse transcription–PCR (RT-qPCR), we performed a selection of the most suitable reference genes for the environmental conditions applied here (for more details, see the Materials and Methods). The selection of putative reference genes was based on previous studies in *Arabidopsis thaliana* (Hong et al. 2010). The putative reference genes included Mu1-adaptin (*ap1m1*), eukaryotic translation elongation factor 1α (*eef1*), the protein phosphatase 2A subunit B (*pp2a*), a TIP41-like protein (*tip41*), β-tubulin 1 (*tub1*), a ubiquitin ligase (*ubc8*) and commonly used reference genes, such as *actin* (*act*) and a receptor of the activated protein kinase C (*cblp*) (Table 1).

**Table 1:** Information on the accession numbers, function and primers for eight potential reference genes and the two genes of interest: *flvA* and *flvB*

Symbol	Accession No.	Description	Primer (5′→3′)	Amplicon length (bp)
*act*	Cre13.g603700	Actin	for-CGCTGGAGAAGACCTACGAG	134
			rev-CGCTGGAGAAGACCTACGAG	
*ap1m1*	Cre06.g262250	Mu1-Adaptin	for-ATGGTGGATGTGTTCAAGCA	145
			rev-TGTACTCGGCCAGGATTTTC	
*cblp*	g6364	Receptor of activated protein kinase C	for- CTTCTCGCCCATGACCAC	105
			rev- CCCACCAGGTTGTTCTTCAG	
*eef1*	Cre12.g498600	Eukaryotic translation elongation factor 1α	for-GAGCTGGAGAAGCTGAAGGA	164
			rev-GCGTCGATGATGGTGTAGTG	
*pp2a*	g1227	Ser/Thr-specific protein phosphatase 2A, subunit B	for-GACTATGATGGCCTGCACCT	230
			rev-CACCGGACCTTGTTGATTTT	
*tip41*	Cre17.g734150	TIP41-like protein	for-GTCTTCCACCTGAACCGTGT	209
			rev-GGTGTGGTGAAGGTCCAGTC	
*tub1*	Cre12.g542250	β-Tubulin 1	for-GCCCTGTACGACATCTGCTT	75
			rev-GCTGATCAGGTGGTTCAGGT	
*ubc8*	Cre03.g159200	Ubiquitin-protein ligase	for-GGATCCGGAACTTCACAAGA	184
			rev-CAGCAGGTGGCAGTACAGAA	
*flvA*	Cre12.g531900	Flavodiiron protein A	for-CAAGTGGTGCTGTCCAACC	246
			rev-GGAGAAGAGCTTGGAGCTGA	
*flvB*	Cre16.g691800	Flavodiiron protein B	for-CGAGGACACCATCACCATC	95
			rev-GGTAGGCGTTGTAGGTGGTG	

**Fig. 1:**
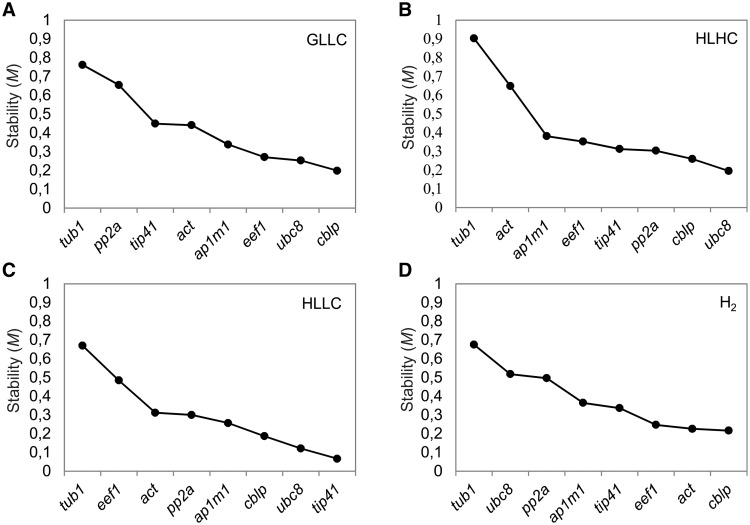
The average expression stability (*M*) of eight tested potential reference genes. *Chlamydomonas reinhardtii* cultures were exposed for 0, 2, 12 and 24 h to GLLC (A), HLHC (B) and HLLC (C). The samples during sulfur deprivation for H_2_ photoproduction (D) were collected at 0, 2, 40 and 150 h from the beginning of sulfur deprivation.

Under growth light and low (ambient level) CO_2_ (GLLC) conditions, the *cblp* and *ubc8* genes showed the lowest *M* values and, therefore, the highest expression stabilities (Fig. 1A). Under the same conditions, *tub1* and *pp2a* were the least stable genes. For cultures under high light and high CO_2_ (HLHC) conditions, *ubc8* and *cblp* were the most stable, while *tub1* and *act* were the least stable genes (Fig. 1B). Under the combined stress of high light and low CO_2_ (HLLC), the most stable reference genes were *tip41*, *ubc8* and *cblp*, whereas *tub1* and *eef1* could not be considered stable (Fig. 1C). The *cblp*, *act* and *eef1* genes showed the most stable expression pattern under the long-term H_2_ photoproduction condition caused by sulfur deprivation, while *tub1* and *ubc8* were the least stable genes (Fig. 1D).

Consequently, *cblp* and *ubc8* were considered suitable reference genes under GLLC, HLHC and combined HLLC conditions. In line with these results, the *cblp* gene has previously been used as a reference gene in different studies of *C. reinhardtii* (Mus et al. 2007, Brzezowski et al. 2012, Pape et al. 2012). The least stable gene in GLLC, HLHC and HLLC cultures was *tub1*, which also corresponds well to previous results (Hong et al. 2010, Rosic et al. 2011, Liu et al. 2012). The *cblp* and *act* genes were selected as suitable reference genes for sulfur deprived experiments.

### Expression of FLVA and FLVB under different environmental conditions

**Fig. 2:**
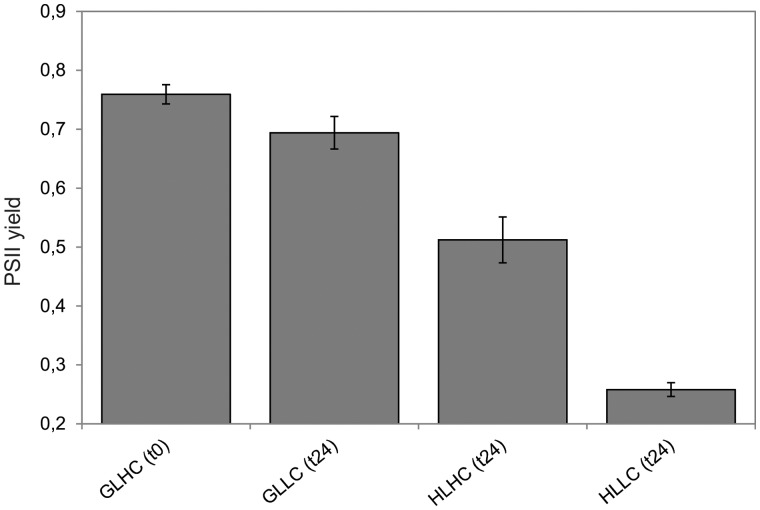
The effective PSII yield monitored directly (*t*0) and 24 h after the shift from GLHC to GLLC, HLHC or HLLC. The values are the mean of three biological replicates (±SD).

To evaluate the possible physiological role of FDPs in autotrophically-grown *C. reinhardtii*, *flvA* and *flvB* transcript levels were studied during the shift from growth light and high CO_2_ (GLHC) condition to GLLC, HLHC and, finally, to HLLC condition. The shift of the cultures from moderate growth light to high light and/or low CO_2_ should lead to a more reduced state of the photosynthetic electron transport chain in the cells. Indeed, the effective yield of PSII dramatically decreased after the 24 h shift from GLHC (0.76) to HLLC (0.26), whereas the shifts to GLLC or HLHC demonstrated a somewhat milder effect (0.69 and 0.51, respectively) on the photosynthetic activity (Fig. 2).

**Fig. 3:**
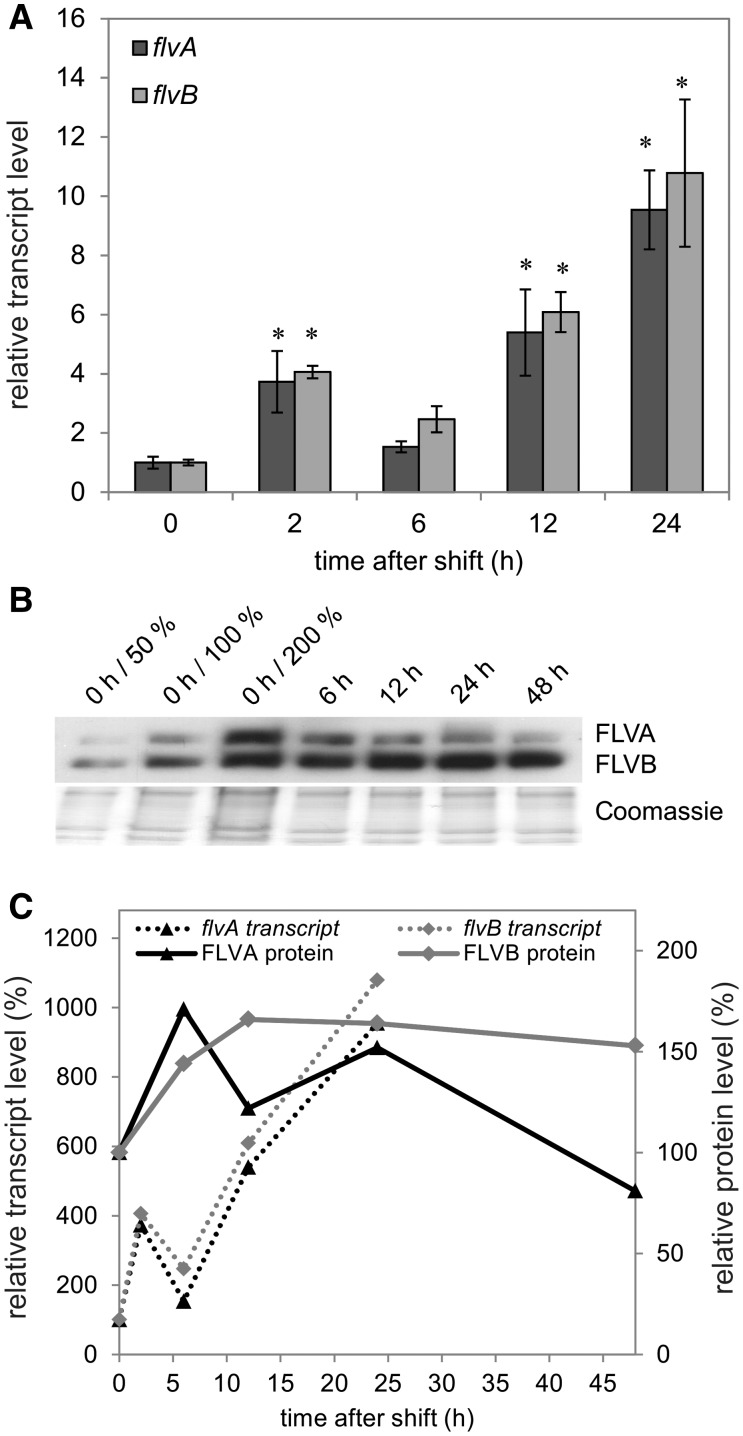
Changes in *flv* transcript (A) and FDP protein (B) levels upon the shift from GLHC to GLLC. RNA was isolated after 0, 2, 6, 12 and 24 h. The values are the mean of three biological replicates (± SD). The significance was evaluated with Student’s *t*-test (an asterisk represents *P* ≤ 0.05). The FLVA and FLVB protein accumulation was probed at 0, 6, 12, 24 and 48 h after the shift by a specific antibody. Correlation between transcript (dashed line) and protein (solid line) accumulation level (C). The protein levels were determined by densitometric analysis of three Western blots, performed with Gene Tools (Perkin Elmer).

The shift of the cells from GLHC to GLLC conditions led to an approximately 4-fold increase in *flvA* transcript abundance after 2 h and up to approximately 9-fold after 24 h acclimation to GLLC (Fig. 3A). The *flvB* transcript level was also significantly up-regulated under the GLLC condition (Fig. 3A), approximately 4-fold after 2 h and approximately 11-fold after 24 h.

In order to corroborate the transcript level results obtained by RT–qPCR on a protein expression level, we generated an antibody against FLVB. The antibody raised against FLVB showed two strong bands around 70 and 58 kDa. The respective bands were cut from the SDS–PAGE gel and submitted to liquid chromatography-tandem mass spectrometry (LC-MS/MS) for further analysis of the functionality of the antibodies. FLVB was identified at approximately 58 kDa and FLVA at approximately 70 kDa. This correlated well with the two strongest bands detected by immunoblotting. The analysis of FDPs under the different environmental conditions demonstrated up-regulation (∼160%) of the FLVA and FLVB proteins 6–48 h after the shift from GLHC to GLLC (Fig. 3B, C).

**Fig. 4:**
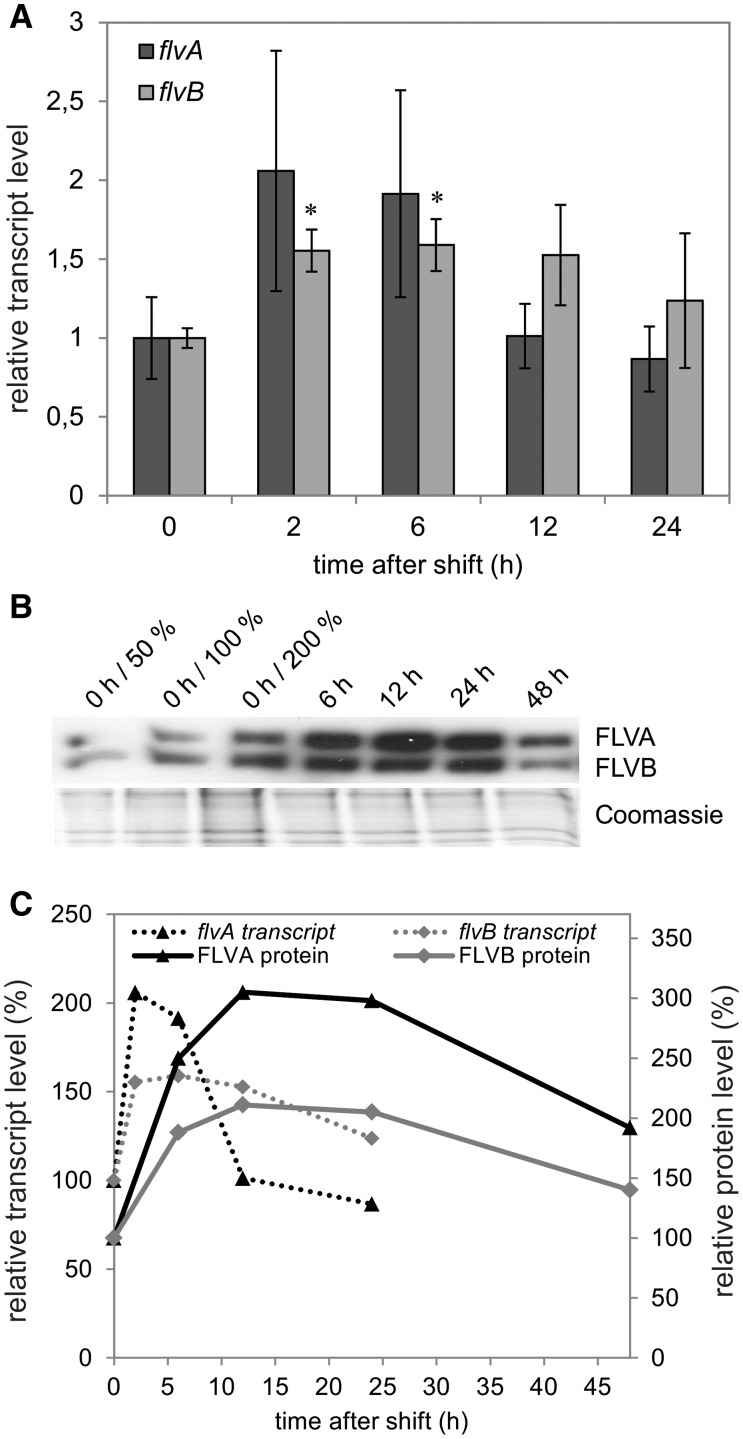
Changes in *flv* transcript (A) and FDP protein (B) levels upon the shift from GLHC to HLHC. RNA was isolated after 0, 2, 6, 12 and 24 h. The values are the mean of three biological replicates ( ± SD). The significance was evaluated with Student’s *t*-test (an asterisk represents *P* ≤ 0.05). The FLVA and FLVB protein accumulation was probed at 0, 6, 12, 24 and 48 h after the shift by a specific antibody. Correlation between transcript (dashed line) and protein (solid line) accumulation level (C). The protein levels were determined by densitometric analysis of three Western blots, performed with Gene Tools (Perkin Elmer).

The shift from growth (GLHC) to high light (HLHC) conditions resulted in a small but significant increase in the transcript abundance of *flvB* (∼1.5-fold) (Fig. 4A). This was reflected at the protein level by immunoblotting experiments showing an increase in the FLVB protein (∼210%) during the first 24 h of the shift to HLHC (Fig. 4B, C). Despite the absence of statistically significant change in *flvA* transcript levels (Fig. 4A), at the protein level FLVA was strongly up-regulated (∼300%) during the first 24 h after the shift to HLHC (Fig. 4B, C). However, 48 h after the shift to HLHC, the abundance of FDPs decreased to approximately 200% for FLVA and to 140% for FLVB.

**Fig. 5:**
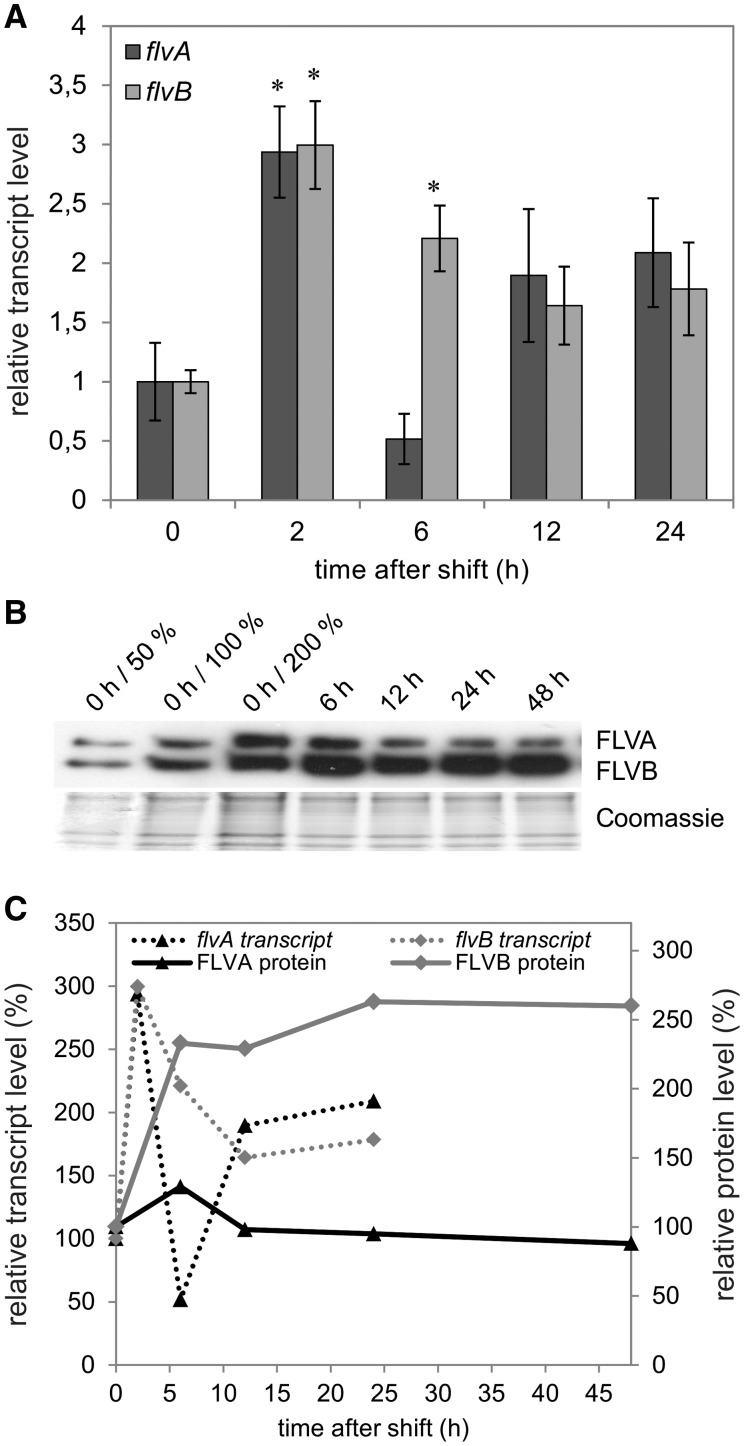
Changes in *flv* transcript (A) and FDP protein (B) levels upon the shift from GLHC to HLLC. RNA was isolated after 0, 2, 6, 12 and 24 h. The values are the mean of three biological replicates (± SD). The significance was evaluated with Student’s *t*-test (an asterisk represents *P* ≤ 0.05). The FLVA and FLVB protein accumulation was probed at 0, 6, 12, 24 and 48 h after the shift by a specific antibody. Correlation between transcript (dashed line) and protein (solid line) accumulation level (C). The protein levels were determined by densitometric analysis of three Western blots, performed with Gene Tools (Perkin Elmer).

The combined stress caused by the shift from GLHC to HLLC led to a small (∼3-fold), but significant, up-regulation of both *flvA* and *flvB* transcripts 2 h after the shift (Fig. 5A). The immunoblot analysis showed a slight up-regulation of FLVA (∼130%) and a strong up-regulation of FLVB (∼260%) 6 h after the shift (Fig. 5B, C). Furthermore, after 48 h at HLLC conditions, the FLVB level did not change further, while the FLVA content decreased to initial levels.

### Long-term H_2_ photoproduction and FDPs

**Fig. 6:**
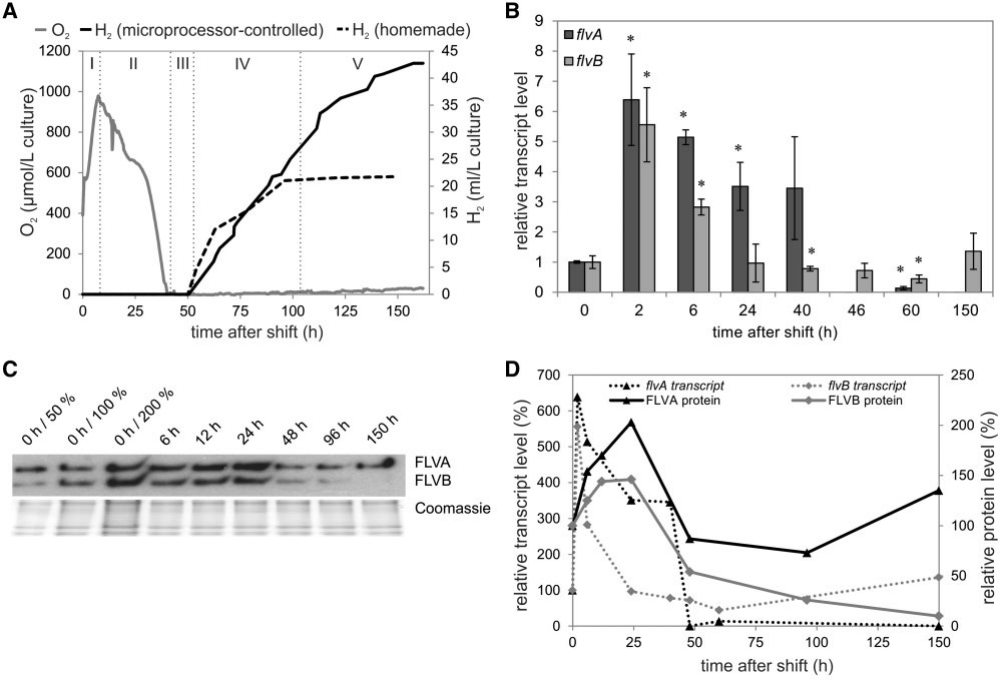
Phases during sulfur deprivation in *C. reinhardtii* cultures in a microprocessor-controlled photobioreactor system (A). H_2_ photoproduction in *C. reinhardtii* cultures was induced by transferring the cells at 0 h into TAP-S medium and the produced H_2_ (full line, microprocessor-controlled photobioreactor system; dashed line, home-made photobioreactor) was gathered by water displacement in the upside-down graduated cylinder filled with water. The O_2_ level was monitored by the microprocessor-controlled photobioreactor system. The relative transcript levels of *flvA* and *flvB* during the shift to H_2_ photoproduction conditions are shown for 0, 2, 6, 24, 40, 46, 60 and 150 h after the beginning of sulfur deprivation (B). The values are the mean of three biological replicates (± SD) and the significance was evaluated with Student’s *t*-test (an asterisk represents *P* ≤ 0.05). The protein levels during H_2_ photoproduction are shown for 0, 6, 12, 24, 48, 96 and 150 h after the shift to TAP-S medium (C). Correlation between transcript (dashed line) and protein (solid line) accumulation level (D). The protein levels were determined by densitometric analysis of three Western blots, performed with Gene Tools (Perkin Elmer).

Next, we investigated the gene transcription and protein expression levels of FDPs throughout acclimation to the H_2_ photoproduction condition, which was triggered by applying a two-stage sulfur deprivation protocol (Melis et al. 2000). Initial experiments were performed in a photobioreactor system that allowed the detection of H_2_ photoproduction yields and continuous monitoring of dissolved O_2_ levels in the media containing sulfur-deprived *C. reinhardtii* cells (Fig. 6A). During acclimation to sulfur deprivation, *C. reinhardtii* cultures pass through five consecutive phases: photosynthetic (I), O_2_ consumption (II), anaerobic (III), H_2_ production (IV) and termination (V) (Kosourov et al. 2002). As shown in Fig. 6A, after the transfer to sulfur deprived photoheterotrophic conditions, the *C. reinhardtii* cells continued to evolve O_2_ intensively during the first 10 h (phase I). In the second phase of sulfur deprivation, the cells undergo strong metabolic changes, PSII activity drops down strongly and respiratory activity increases, inducing a transition to anaerobic conditions. This phase is followed by a complete anaerobic phase (Fig. 6A, phase III), where two [Fe–Fe]-hydrogenases are expressed, finally leading to H_2_ photoproduction (phase IV). Similar experiments were also performed in sealed flasks, where H_2_ photoproduction was monitored regularly (Fig 6A, dashed line) and samples were collected from different time points of sulfur deprivation for further investigation by RT–qPCR and Western blotting experiments.

RT–qPCR experiments demonstrated that the transcript levels of *flvA* and *flvB* were significantly up-regulated (∼6.5-fold and ∼5.5-fold, respectively) 2 h after the shift to TAP-S (Tris-acetate-phosphate without sulfur) medium (Fig. 6B), when PSII is still active and a significant increase in the level of dissolved O_2_ is observed (Fig. 6A). The *flvA* transcript level remained up-regulated (∼3.5-fold) while the *flvB* level decreased to approximately 0.7-fold of the initial level at 40 h of sulfur deprivation, when complete anaerobiosis was established and H_2_ photoproduction began. During the H_2_ photoproduction phase (46–60 h after the shift), when cultures had established a long-term anaerobiosis in the medium (Fig. 6A), both *flvA* and *flvB* transcript levels were significantly down-regulated to 0.1-fold (or not detectable) and approximately 0.5-fold, respectively (Fig. 6B). During the termination phase, 150 h after the transfer to sulfur deprivation conditions when cultures cease producing H_2_ and, instead, start to show residual O_2_ evolution activity (Fig. 6A), *flvB* returned to its initial transcript level and *flvA* was no longer detectable.

Immunoblotting revealed a strong up-regulation in the amounts of FLVA (∼200%) and FLVB (∼150%) proteins from 6 to 24 h after the shift to TAP-S medium (Fig. 6C, D). The later time points showed that the FLVA protein had returned to its initial level by the start of H_2_ photoproduction and FLVB declined until it was undetectable at the end of the experiment. Although the protein levels did not strictly follow the trend of the transcript abundance towards the termination phase, it is clear that the FLVA and FLVB proteins were up-regulated during the photosynthetic phase of adaption to sulfur deprivation and remained high until anaerobiosis was established in the culture.

## Discussion

### Analysis of putative reference genes

As a first approach to obtain information about the function of FDPs in *C. reinhardtii*, we applied RT–qPCR to determine the response of *flv* transcript levels to varying environmental conditions. The determination of appropriate reference genes for each organism under particular environmental conditions is crucial to employing the correct normalization strategy to transcript analysis (Huggett et al. 2005, Guenin et al. 2009). In this study, we analyzed several candidate reference genes, and the *cblp* and *ubc8* genes were determined to be the most suitable for the interpretation of the transcript data obtained after the shift of algal cultures from high CO_2_ and standard growth light conditions to low CO_2_ and/or high light conditions (Fig. 1A–C).

The situation was different when the sulfur deprivation protocol was applied (Melis et al. 2000) for the initiation of long-term H_2_ photoproduction. Under nutrient deprivation conditions, the sealed algal cultures pass through several physiological stages, resulting in massive changes in cellular metabolism from oxygenic photosynthesis to anaerobic photo-fermentation (Atteia et al. 2013, Catalanotti et al. 2013, Allahverdiyeva et al. 2014). In this case, the *cblp* and *act* genes were the most stable reference genes (Fig. 1D). This study confirmed that there are no universal reference genes, and the choice of appropriate reference genes varies depending on the environmental conditions and the nature of the analyzed target genes.

### FDPs possibly work as an alternative electron sink in *C. reinhardtii*

FDPs are known to function in alternative electron transport routes in cyanobacteria (reviewed in Allahverdiyeva et al. 2015a, Allahverdiyeva et al. 2015b). The presence of homologs of the genes coding for FDPs in *C. reinhardtii* suggests a possible involvement of their products in photosynthetic electron transport. However, the function of FDPs in *C*. *reinhardtii* has not been addressed thoroughly and needs to be elucidated. RT–qPCR and Western blot analysis demonstrated that *flvA* and *flvB* were significantly up-regulated on both the transcript and protein levels after the change in CO_2_ (shifting the cultures from HC to LC) (Figs. 3, 5) and/or light (shifting the cultures from GL to HL) regimes (Figs. 4, 5). The strongest up-regulation of the FLVA protein was observed after the shift to high light, whereas the FLVB protein was up-regulated under all three different environmental conditions tested in the present study.

The treatment of cells with both high light and/or limited CO_2_ concentrations led to a decrease in photosynthetic activity (Fig. 2). The exposure of cells to high light causes an increase in NAD(P)H levels, while the lower CO_2_ availability led to a higher ATP demand (Kramer and Evans 2011). During evolution, photosynthetic organisms have developed sophisticated mechanisms to dissipate excess reducing power in harmless ways and to balance possible mismatches in production and demand of ATP and NAD(P)H, the ratio of which changes upon environmental cues through the regulation of linear and alternative electron transport pathways (Peltier et al. 2010, Cardol et al. 2011, Kramer and Evans 2011). Recent studies with cyanobacteria have demonstrated the function of FDPs as powerful electron sinks under stress conditions: Flv2 and Flv4 are strongly up-regulated and involved in the photoprotection of PSII under ambient CO_2_ and high light conditions (Zhang et al. 2009, Zhang et al. 2012, Bersanini et al. 2014), whereas Flv1 and Flv3 proteins can release electron pressure after PSI, thus safeguarding PSI under fluctuating light intensities (Allahverdiyeva et al. 2013). Flv1 and Flv3 proteins act as a strong electron sink, redirecting about 20–60% of electrons originating from PSII to O_2_ during illumination under air-level CO_2_ and under strong Ci deprivation, respectively (Allahverdiyeva et al. 2011). Importantly, O_2_ photoreduction by the FDP pathway generates water without the formation of reactive oxygen species (ROS) (Vicente et al. 2002), thus also contributing to ATP synthesis.

The data obtained with *C. reinhardtii flvA* and *flvB* genes strongly resembles the gene expression pattern of *Anabaena flv1A* and *flv3A*, where both genes were strongly up-regulated at low CO_2_ and moderately up-regulated at high light conditions (Ermakova et al. 2013). Moreover, accumulation of the *flv3* transcript and a strong up-regulation of the Flv3 protein have been observed in *Synechocystis* cells under low CO_2_ conditions, whereas the *flv1* and *flv3* transcripts in *Synechocystis* did not show a remarkable induction under high light conditions (Zhang et al. 2009). The limited information about the response of *flv1* transcripts to different environmental cues is probably due to low transcript abundance of this gene in *Synechocystis* (Zhang et al. 2009, Allahverdiyeva et al. 2015a).

A recent study showing an up-regulation of FLVA and FLVB proteins in the *pgrl1* mutant of *C. reinhardtii* under low CO_2_ as well as high light conditions indicates that FDPs could function as an electron valve to compensate for the lack of, or impaired, cyclic electron flow (Dang et al. 2014). Interestingly, under the combined HLLC stress condition used in that study, the up-regulation of FDPs in the *pgrl1* mutant was transient and disappeared after 48 h. Instead, the elevated H_2_O_2_ level indicated a replacement of the FDP pathway by the true Mehler reaction and the formation of ROS (Dang et al. 2014). Similarly, our results with *C. reinhardtii* wild type during HLLC stress showed an up-regulation of both FDPs during the first 48 h (Fig. 5). This implies an important function for these proteins upon changes in environmental conditions. Our expression analysis of FLVA and FLVB, together with previous results, suggests that FDPs in *C. reinhardtii* also play an important role as alternative electron sinks in order to prevent redox poise at the photosynthetic electron transport chain.

The possible electron donor of FDPs in *C. reinhardtii* is not known yet. Based on *in vitro* studies on recombinant *Synechocystis* Flv3 proteins, it was concluded that FDPs function as an NAD(P)H-O_2_-oxidoreductase (Vicente et al. 2002). However, this is not the case for *Synechocystis* Flv2 and Flv4 proteins functioning at the PSII acceptor side. Recently, ferredoxin 1 (FDX1) was found to interact with FLVB, thus opening up a new discussion about the possibility of FDX1 as an electron donor to the FDPs proteins in *C. reinhardtii* (Peden et al. 2013).

### FDPs participate in photosynthetic acclimation of *C. reinhardtii* to sulfur deprivation

During acclimation to sulfur deprivation, algae experience a strong metabolic shift from oxygenic photosynthesis, where CO_2_ is assimilated and starch accumulated, towards anaerobic photo-fermentation, where starch reserves are metabolized to produce ATP and NAD(P)H. The anaerobic re-oxidation of NAD(P)H involves several fermentative pathways that produce organic acids (acetate, formate, lactate, malate and succinate), ethanol, H_2_ and CO_2_ (reviewed in Atteia et al. 2013, Catalanotti et al. 2013). Some enzymes of fermentative metabolism, such as the [Fe–Fe]-hydrogenases and pyruvate formate-lyase, are sensitive to O_2_ remaining in the chloroplast (Atteia et al. 2013).

The acclimation to sulfur deprivation that triggers H_2_ photoproduction in algae can be divided into several phases (Kosourov et al. 2002) (Fig. 6A). During the photosynthetic stage (phase I) of acclimation to sulfur deprivation (0 to ∼10 h) the O_2_ concentration in the bioreactor rises until respiratory processes take over (phase II). Anaerobiosis is established at approximately 40 h after the shift to sulfur deprivation (phase III). The up-regulation of both FLVA and FLVB proteins demonstrates a correlation with the presence of O_2_ in the culture, with the maximum FDP amount observed approximately 24 h after the shift (Fig 6C, D). The FDP up-regulation during the photosynthetic and respiratory phase of H_2_ photoproduction indicates that O_2_ photoreduction via FDPs is important in the acclimation to these conditions. It has been postulated that the decrease of O_2_ after a shift to sulfur deprived medium is mainly due to an increase in mitochondrial respiration (Melis et al. 2000, Melis 2007, Ghirardi et al. 2010). Our results indicate that FDPs contribute to the establishment of anaerobiosis by functioning in light-induced O_2_ uptake. The increased levels of FDPs in the chloroplast during the first phases of sulfur deprivation may accelerate the establishment of anaerobiosis and therefore help to ensure the function of the fermentative pathways within a shorter time period. In the later phase IV, while H_2_ is produced in anaerobiosis, the FDPs are down-regulated (Fig. 6).

Taken together, we propose that FDPs in *C. reinhardtii* function as an alternative electron sink during oxygenic photosynthesis by actively assisting to decrease the O_2_ level inside the chloroplast at the onset of anaerobiosis and are replaced by [Fe–Fe]-hydrogenases later on, when anaerobiosis is fully established. In both cases, FDPs and [Fe–Fe]-hydrogenases support electron flow in thylakoids for the production of ATP at the expense of reducing power accumulated downstream of PSI and, thus, also protect the photosynthetic electron transport chain from over-reduction. This rapid acclimation to anaerobiosis is likely to be advantageous for the soil-dwelling *C. reinhardtii*, which regularly faces anoxic or micro-oxic conditions in nature.

## Materials and Methods

### Strains and culture conditions

The wild-type *C. reinhardtii*, strain CC406, was maintained photoheterotrophically in TAP medium (Gorman and Levine 1965) at ambient air under a continuous light intensity of 50 µmol photons m^−2 ^s^−1^ photosynthetically active radiuiation (PAR) under agitation (90 r.p.m.) at 25°C. For preparing experimental *C. reinhardtii* cultures, the cells were harvested at OD_750_ = approximately 1.2, transferred to high salt medium (HSM; Sueoka 1960), diluted to OD_750_ = approximately 0.2 and cultivated photoautotrophically by bubbling the cultures with sterile air containing 3% CO_2_ (high CO_2_, HC) under a continuous light intensity of 50 µmol photons m^−2 ^s^−1^ PAR (GL), at 25°C for 48 h. To perform the shift to different environmental conditions, the cells were harvested at OD_750_ = 1.2 by centrifugation (2,500 r.p.m., 2 min), resuspended in fresh HSM and adjusted to OD_750_ = 1.0. High light conditions were achieved by illuminating the cells at 150 µmol photons m^−2 ^s^−1^ with 3% CO_2_ (HLHC) and low CO_2_ conditions were obtained by bubbling the cells with ambient air under 150 µmol photons m^−2 ^s^−1^ (HLLC), or under standard growth light, 50 µmol photons m^−2 ^s^−1^ (GLLC). Cells for transcript analysis were collected at 0, 2, 6, 12 and 24 h, and for protein analysis at 0, 6, 12, 24 and 48 h after the shift and stored at –80°C until further use.

### Chl fluorescence measurement

The Chl *a* fluorescence was analyzed with a Dual-PAM-100 fluorometer (Walz). The cultures were adjusted to a final Chl (a+b) concentration of 10 µg ml^−1^. Red actinic light (630 nm) intensity of 54 µmol photons m^−2 ^s^−1^ (cells grown under standard growth light) or 217 µmol photons m^−2 ^s^−1^ (cells treated with high light) was applied. The PSII effective yield was calculated as Y(II) = (*F*_m_′ – *F*)/*F*_m_′ after illumination of the cells with an actinic light for 5 min. A saturating pulse (4,000 µmol photons m^−2 ^s^−1^, 500 ms) was fired to probe *F*_m_′, the maximum fluorescence level under the actinic light. *F* is the steady-state fluorescence level under actinic light.

### H_2_ photoproduction

For the long-term H_2_ photoproduction, a sulfur deprivation protocol was applied (Melis et al. 2000). Cultures grown in standard TAP medium (Gorman and Levine, 1965) under ambient CO_2_ and continuous light intensity of 50 µmol photons m^−2 ^s^−1^ PAR were harvested by centrifugation at a cell density of approximately 25 µg ml^−1^ Chl (a+b) and transferred into TAP medium without sulfur (TAP-S). After a series of centrifugations (twice at 2,500 r.p.m. for 2 min) and re-suspensions in TAP-S medium, the cells were adjusted to a Chl (a+b) concentration of 20 µg ml^−1^. Home-made cylindrical photobioreactors with an inner diameter of 60 mm were filled with 550 ml of culture, placed under continuous illumination of 75 µmol photons m^−2 ^s^−1^ PAR from cool white fluorescent lamps (Mitsubishi/Osram) at 25°C and kept sealed with threaded rubber stoppers and attached tubing for gas collection. H_2_ production was monitored by collecting the gas in an upside-down graduated cylinder filled with water. For transcriptional analysis, cells were taken at 0, 2, 6, 24, 40, 46, 60 and 150 h after the shift to TAP-S medium. Cells for the protein analysis were collected at 0, 6, 12, 24, 48, 96 and 150 h after the shift. For continuous monitoring of the O_2_ level in the cultures, the experiment was repeated in a microprocessor-controlled photobioreactor system, described in Tsygankov et al. (2006).

### RNA extraction

Total RNA was extracted using TRIsure (Bioline) reagent. The cells were broken via heating at 65°C. RNA was further purified by extraction with phenol/chloroform/isoamylalcohol (25 : 24 : 1) and precipitated by isopropanol, followed by removal of genomic DNA (Ambion Turbo DNase kit) with 0.5 µl of DNase (2 U µl^−1^). The RNA concentration was determined using a NanoDrop ND-1000 spectrophotometer (Thermo Scientific) and the quality was checked by RNA-gel electrophoresis.

### cDNA synthesis

Purified RNA (2 µg) was used for cDNA synthesis. Reverse transcription was performed with poly(dT)_(20)_ primers and SuperScript III Reverse Transcriptase (Invitrogen) according to the manufacturer’s protocol. Synthesized cDNA was diluted 5-fold and used as a template for RT–qPCR.

### RT–qPCR

RT–qPCR was performed with a Bio-Rad IQ5 system using iQ SYBR Green Supermix (Bio-Rad) in 96-well plates. The PCR protocol was 3 min initial denaturation at 95°C, followed by 50 cycles of 95°C for 15 s, 60°C for 30 s and 72°C for 35 s. At the end, a melting curve was performed. Relative changes in the gene expression level were calculated using the qbase^+^ software by Biogazelle.

### Selection of putative reference genes and primer design

For calculation of the expression levels of target transcripts, most popular methods, such as the 2^–ΔΔCT^ method (Schmittgen and Livak 2008), apply reference genes for normalization. Eight putative reference genes (Table 1) were selected based on previous studies of reference genes in *A. thaliana* (Hong et al. 2010) and some commonly used reference genes in *C. reinhardtii*. The specific primers were designed by using Primer3plus software (http://www.bioinformatics.nl/cgi-bin/primer3plus/primer3plus.cgi) and are shown in Table 1.

For correct comparison, it is recommended to select reference genes with a similar expression level compared with the target genes (Hruz et al. 2011). As shown in Supplementary Fig. 1, the Cq values of the studied putative reference genes varied between 24 and 35, and those of the target genes varied between 28 and 40. Each putative reference gene showed a relatively stable expression, with Cq values that differed in just 1–2 cycles. The stability of the putative reference genes was validated by the geNorm algorithm (Vandesompele et al. 2002) included in the qbase^+^ software. The minimum number of reference genes needed for normalization was determined by calculation of the pairwise variation (*V*). A threshold of 0.15 was applied and the lowest *V* values were obtained at *V*_2/3_, which means that a minimum of 2–3 reference genes are sufficient for normalization of the expression data under all studied conditions (Supplementary Fig. 2).

### Protein analysis

Total protein extracts were isolated by resuspending the sample cell pellet in lysis buffer (50 mM Tris pH 8, 2% SDS, 10 mM EDTA, protease inhibitors; Sigma) and freezing. After thawing the samples, the total protein extracts were separated by 14% SDS–PAGE without urea, transferred to a polyvinylidene difluoride membrane (Millipore) and blocked with 5% blotting grade blocker (Bio-Rad). The samples were loaded on an equal protein basis determined with a Direct Detect™ Spectrometer (Millipore) and visualized with Coomassie brilliant blue (Bio-Rad). The FLVA and FLVB proteins were detected using a purified rabbit antibody prepared against an FLVB peptide antigen mix (CKVVIAESYGGRDEP and CARKKAAMSGEVAKA) conjugated with keyhole limpet hemocyanin. The high homology between FLVB and FLVA allows this antibody to recognize both proteins.

The specificity of the antibody was verified via LC-MS/MS. As a secondary antibody, anti-rabbit horseradish peroxidase (HRP) was used and visualized with ECL. The protein levels were determined by densitometric ananlysis of three Western blots, performed with Gene Tools (Perkin Elmer)

### Identification of proteins by LC-MS/MS

Samples for LC-MS/MS analysis were prepared according to the protocol of Shevchenko et al. (1996). Silver-stained protein bands were excised from the SDS–PAGE gel, reduced, alkylated and in-gel digested with Trypsin Gold (Promega). The peptides were extracted by repeated incubation with 5% formic acid and50% acetonitrile, lyophilized and desalted on C18 resin. Samples were analyzed by LC-MS/MS using a QExactive mass spectrometer (Thermo Fisher Scientific, Inc.) connected in line with an Easy-nLC II HPLC system (Thermo Fisher Scientific, Inc.). Peptides were dissolved in 18 µl of 2% formic acid. From the sample, 5 µl were loaded onto a pre-column (2 cm × 100 µm inner diameter) packed with a Magic C18 AQ 200-Å resin (Michrom Bioresources) and subjected to reverse-phase chromatography on a 15 cm × 75 µm inner diameter nanoscale LC column packed with the same resin. A gradient of 2–40% acetonitrile in 0.2% formic acid was applied for 28 min followed by a gradient of 40–100% acetonitrile in 0.2% formic acid for 2 min with a flow rate of 300 nl min^−1^. MS data acquisition of positively charged precursor ions was performed in a data-dependent mode, with the 10 most intensive ions sent to MS/MS analysis in each duty cycle. The data were processed with the Mascot search engine (version 2.4; Matrix Science) through the Protein Discoverer software, version 1.4 (Thermo Fisher Scientific, Inc.). Database searches were performed against a database of *C. reinhardtii* proteins supplemented with sequences of common protein contaminants and with a reverse decoy database. The search criteria allowed for one miscleavage of trypsin, oxidation of methionine, and 5 and 10 p.p.m. mass accuracies for MS and MS/MS modes, respectively.

## Supplementary data

Supplementary data are available at PCP online.

## Funding

This research was financially supported by the Kone foundation [to Y.A.]; the Academy of Finland and Russian Academy of Science [Research exchange program (2011–2013) mobility (grant # 267409 to Y.A.)]; the Academy of Finland [FCoE program (271832 to E-M.A.)]; the Maj and Tor Nessling Foundation [2014050 to S.K.] the Nordic Energy Research AquaFEED project.

## Supplementary Material

Supplementary Data

## Abbreviations

FDP: flavodiiron protein
FDX1: ferredoxin 1
GL: growth light
HC: high CO_2_
HL: high light
LC: low CO_2_
LC-MS/MS: liquid chromatography-tandem mass spectrometry
PAR: photosynthetically active radiation
ROS: reactive oxygen species
RT–qPCR: real-time quantitative reverse transcription–PCR
TAP-S: Tris-acetate-phosphate without sulfur
