# Supplementary material for: Chlamydomonas Flavodiiron Proteins Facilitate Acclimation to Anoxia During Sulfur Deprivation
Source: Plant Cell Physiol. 2015 Jun 10;56(8):1598–607. doi: 10.1093/pcp/pcv085 (PMC4523385; doi:10.1093/pcp/pcv085)
Supplement: Supplementary Data [file supp_56_8_1598__index.html]

Chlamydomonas Flavodiiron Proteins Facilitate Acclimation to Anoxia During Sulfur Deprivation — Chlamydomonas Flavodiiron Proteins Facilitate Acclimation to Anoxia During Sulfur Deprivation — Supplementary Data 

# *Chlamydomonas* Flavodiiron Proteins Facilitate Acclimation to Anoxia During Sulfur Deprivation

## Supplementary Data

files

- Supplementary Data - pdf file
