# Supplementary material for: Chlamydomonas Flavodiiron Proteins Facilitate Acclimation to Anoxia During Sulfur Deprivation
Source: Plant Cell Physiol. 2015 Jun 10;56(8):1598–607. doi: 10.1093/pcp/pcv085 (PMC4523385; doi:10.1093/pcp/pcv085)
Supplement: Supplementary Data [file supp_pcv085_pcp-2015-e-00216-File008.pdf]

Supplemental material

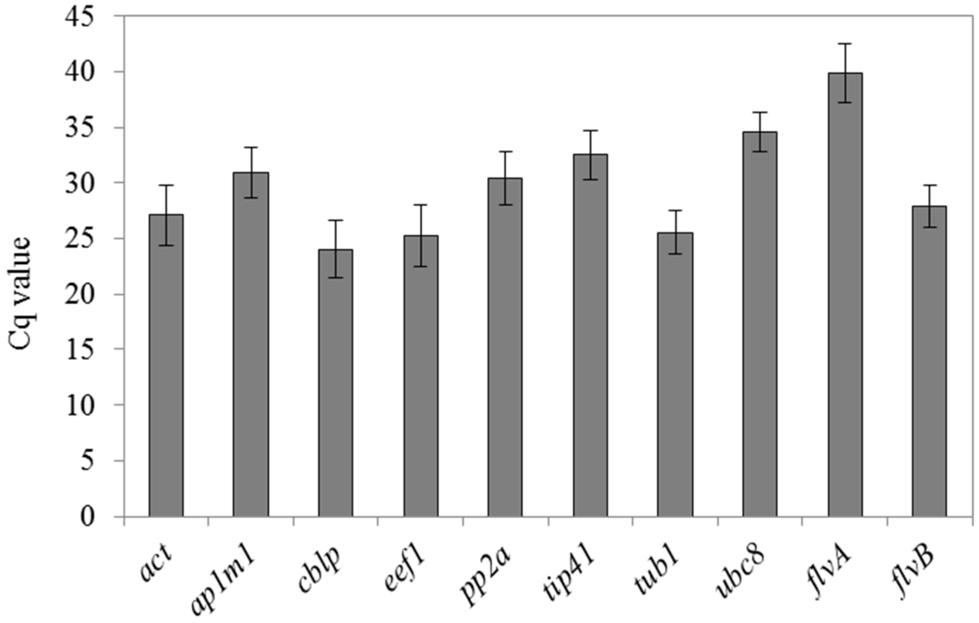

Suppl. Fig. 1: Cq mean values of the tested potential reference genes and the two genes of interest in response to all environmental conditions applied in this work. *C. reinhardtii* cultures were exposed for 0, 2, 6, 12 and 24 h to low carbon (GLLC), high light (HLHC) and combined high light / low carbon (HLLC). The samples for hydrogen production (H_2) conditions were taken at 0, 2, 6, 24, 40, 60 and 150 h. The Cq values are mean of four biological replicates (± SD).

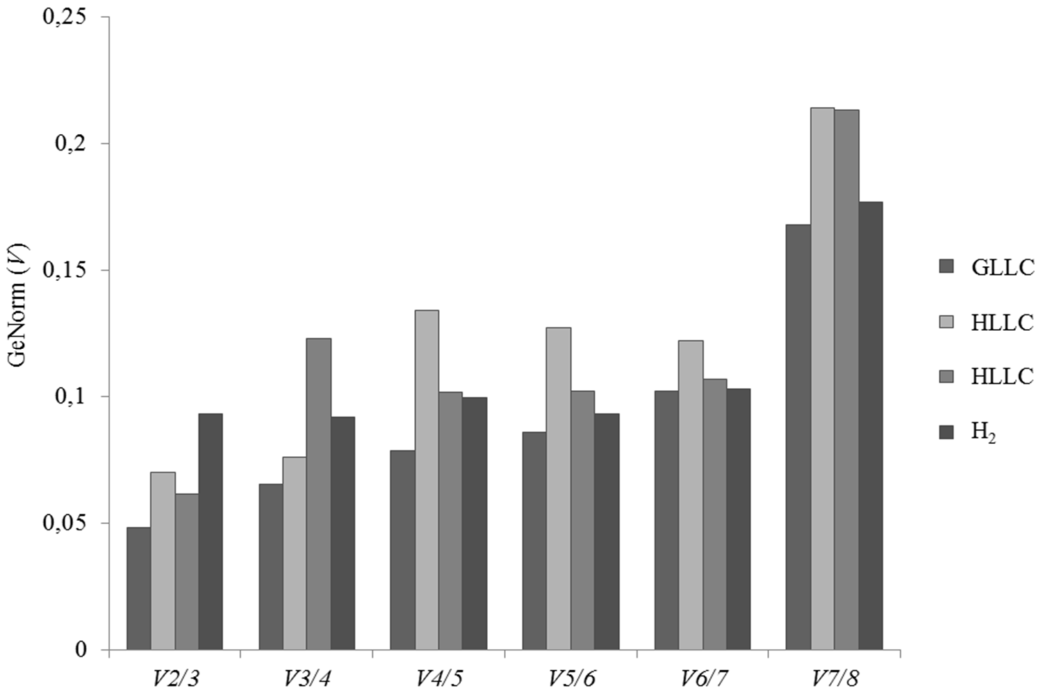

Suppl. Fig. 2: The pairwise variation (*V*) of the eight tested potential reference genes to determine the optimal number of reference genes employed for further normalization upon low carbon (GLLC), high light (HLHC), combined low carbon and high light (HLLC) and hydrogen production (H_2) conditions.
